# The frequency and severity of ultrasound-detected osteoarthritis features in the knees and their associations with pain: Cross-sectional analyses of the Nor-Hand study

**DOI:** 10.1016/j.ocarto.2025.100640

**Published:** 2025-06-05

**Authors:** Caroline H. Dekkerhus, Alexander Mathiessen, Caroline M. Fjellstad, Barbara Slatwkosky-Christensen, Hilde Berner Hammer, Ida K. Haugen

**Affiliations:** a Center for Treatment of Rheumatic and Musculoskeletal Diseases (REMEDY), Diakonhjemmet Hospital, Oslo, Norway; b Institute of Clinical Medicine, University of Oslo, Oslo, Norway

**Keywords:** Osteoarthritis, Knee osteoarthritis, Pain, Ultrasound

## Abstract

**Objective:**

To investigate the frequency and severity of ultrasound-detected osteophytes and synovitis in people with and without knee osteoarthritis (OA), and to explore the association between these ultrasound features and pain.

**Design:**

In the Nor-Hand study, both knees were assessed for osteophytes (0–3 scale, four locations per knee) and grey-scale synovitis (0–3 scale). The frequency and severity of the ultrasound-detected features were compared in individuals with and without knee OA defined by the American College of Rheumatology criteria. Pain was self-reported in each knee (yes/no) and by the Western/Ontario McMaster University index (WOMAC). The associations between ultrasound-detected features and pain were examined by regression analyses adjusted for age, sex, and body mass index.

**Results:**

We analyzed 286 participants. Osteophytes of all sizes were more common in participants with knee OA compared to those without (65.9 ​% vs. 40.8 ​%, p ​< ​0.001). No between-group difference was found for the frequency of any grey-scale synovitis (45.5 ​% vs. 44.7 ​%, p ​= ​0.67), while severe synovitis was more common in those with knee OA. Ultrasound-detected osteophyte sum score, but not synovitis, was associated with WOMAC pain (B ​= ​0.18, 95 ​% CI 0.03–0.32). Osteophytes of all sizes were associated with pain in the same knee with odds ratio (OR, 95 ​% CI) ranging from 1.85 (1.20–2.84) to 9.02 (4.04–20.10). Statistically significant association was found for severe synovitis only (OR ​= ​6.63, 95 ​% CI 2.26–19.43).

**Conclusions:**

Ultrasound-detected osteophytes were prevalent in people with knee OA and were associated with pain. OA pathology in individuals without fulfilling the knee OA criteria may reflect early or subclinical OA.

## 1 Introduction

Knee osteoarthritis (OA) is a common cause of pain, disability, and healthcare utilization, particularly in older adults. Symptomatic knee OA affects approximately 12 ​% of individuals over 60 years [1], and its prevalence continues to rise globally. The main symptoms – pain, stiffness, and reduced function – are well recognized, yet their underlying causes remain incompletely understood.

Traditionally, OA has been assessed on conventional radiographs, but discordance between radiographic severity and knee pain has been widely reported [2]. Nonetheless, when between-person confounding is minimized, a significant relationship between radiographic changes and knee pain emerges, even in early OA [3,4]. In recent decades, the increasing application of modern imaging modalities such as ultrasound and MRI have provided relevant additional diagnostic information on tissue-specific changes that are not depicted by conventional radiography. They support previous histological and molecular evidence of inflammation being an active component of the OA process [5].

Ultrasound, in particular, has emerged as a valuable tool for assessing knee OA. It is a valid, cost-effective, and reproducible method for detecting synovitis, effusion, osteophytes, and meniscal extrusion, with good correlation to MRI and histological findings [[6], [7], [8], [9], [10], [11]]. Despite its advantages, the evidence linking ultrasound findings to clinical symptoms, such as knee pain, remains inconsistent. For example, while some studies have demonstrated positive associations between synovitis and pain [[12], [13], [14], [15]], others have reported no or conflicting associations [[16], [17], [18]]. Variability in study designs, pain assessment tools, and sample sizes contributes to these discrepancies [19].

Importantly, knee OA remains primarily a clinical diagnosis [20], and few studies have evaluated the validity and clinical relevance of ultrasound-detected features against established clinical classification criteria. Additionally, control groups without clinical knee OA have rarely been included [7,21], limiting comparisons.

To address this gap, we aimed to 1) compare the frequency and severity of ultrasound-detected osteophytes and grey-scale synovitis in individuals with and without clinical knee OA, as defined by the American College of Rheumatology (ACR) criteria, and 2) explore the associations between these ultrasound findings and knee pain to assess their construct validity. We hypothesized that both osteophytes and grey-scale synovitis were more frequent in people with clinical knee OA as compared to those without knee OA, and that these OA features were also related to knee pain.

## 2 Methods

### 2.1 Study design

The analyses were based on data from the observational cohort study Nor-Hand. The hospital-based study included 300 men and women (40–70 years) with hand OA determined by clinical examination and/or ultrasound, whereas patients with systemic inflammatory joint diseases, psoriasis, or hemochromatosis were excluded. Detailed information about the inclusion and exclusion criteria was published in the study protocol [22]. All participants gave their informed consent to participate. The Norwegian Regional Committee for Medical and Health Research Ethics approved the study (2014/2057), and the trial is registered at https://clinicaltrials.gov (NCT03083548). Data from the baseline examination in 2016-17 were used in the current analyses.

### 2.2 Questionnaires and clinical examinations

The participants were asked to report their knee/hip pain using the Western Ontario and McMaster Universities Arthritis Index (WOMAC) [23], which was self-administered using a validated Norwegian version. Each of the five questions has 5 response options ranging from none to extreme (0–4), and the sum score for the pain subscale is ranging from 0 to 20. The sum score reflects pain in the knees combined without specifying side. In addition, the patients marked if they had pain in their left or right knee during the last 24 ​h as well as during the previous 6 weeks.

An experienced rheumatologist (BSC) or a trained rheumatology resident examined the presence of clinical knee and hand OA according to the ACR classification criteria [24,25]. Clinical OA of the knee was defined as knee pain and at least three out of six of the following criteria: age >50 years, morning stiffness <30 ​min, crepitus, bony tenderness, bony enlargement, and no palpable warmth. The participants’ weight and height in light indoor clothing and no shoes were measured, and their body mass index (BMI) was calculated.

### 2.3 Ultrasound

A trained medical student (CMF) under supervision by an experienced rheumatologist (HBH) performed the ultrasound examination. Before testing, the medical student went through a detailed training session on normal anatomy and demonstration of OA pathology. The first fourteen patients were assessed together with the experienced rheumatologist. Good agreement was observed, and the medical student completed the rest of the ultrasound examinations independently. The bilateral knees were examined with the patient in a supine position using the ultrasound machine General Electric Logiq E9 (Chicago, Illinois, United States) [22]. The same machine with fixed settings was used throughout the study to standardize the examination. Osteophytes were assessed bilaterally on extended knee with the transducer in a longitudinal orientation on the medial and lateral side of the tibiofemoral joint, examining from anterior to posterior part of the joint space. Osteophytes were evaluated at four locations in each knee (medial and lateral tibia and medial and lateral femur) according to a previously published ultrasound atlas on a 0–3 scale (grade 0 ​= ​normal, 1 ​= ​minor, 2 ​= ​moderate and 3 ​= ​major size of the osteophytes) [26]. The maximum osteophyte score in each location was identified in the right and left knee separately and across both knees. Sum scores for each knee (range: 0–12) and both knees together (range: 0–24) were calculated. Grey-scale synovitis (combined score for hypertrophy and joint fluid) in the suprapatellar recess (0–3 scale) was evaluated with a longitudinal orientation of the probe [27]. A reliability exercise was performed, in which both the medical student and the experienced rheumatologist examined 10 patients. The inter-observer reliability was moderate for both osteophytes and grey-scale synovitis with weighted kappa scores of 0.59 and 0.48, respectively.

### 2.4 Statistics

Patients were divided into two groups based on whether they fulfilled the ACR criteria for clinical knee OA or not. We compared the frequency and the severity of ultrasound findings (osteophytes and grey-scale synovitis) between the two groups using Chi-Square or Mann-Whitney test for categorical variables and sum scores, respectively. We examined whether sum scores or maximum scores of ultrasound-detected OA features in the two knees were associated with WOMAC pain score using linear regression. Logistic regression with generalized estimating equations was used to examine associations between the severity of OA features in each individual knee and pain in the same joint. All analyses were adjusted for age, sex, and BMI. P-values less than 0.05 were considered statistically significant. The sample size of the Nor-Hand study was pragmatically chosen based on previous studies and feasibility, and was not based on formal power calculations. To analyze the data, we used SPSS version 27.0.

## 3 Results

### 3.1 Study population

Among the 300 participants who were enrolled in the Nor-Hand study, 286 participants were included in the present analyses. We excluded 14 participants, of whom 13 reported previous knee prosthesis and 1 reported arthrodesis. Demographic and clinical characteristics of the 286 patients are shown in Table 1. Both osteophytes (mostly small to moderate) and grey-scale synovitis (mostly mild) were commonly found in the study population. Osteophytes were more common in the right (n ​= ​146, 51.0 ​%) than in the left knee (n ​= ​111, 38.8 ​%), while there was no difference between the right (n ​= ​88, 30.8 ​%) and left knee for grey-scale synovitis (n ​= ​92, 32.2 ​%). Pain during the last 24 ​h was frequently reported in both the right (n ​= ​95, 33.2 ​%) and left knee (n ​= ​89, 31.1 ​%). Similar numbers were found for pain during the last 6 weeks, while WOMAC pain score was at the lower end of the scale (Table 1).

**Table 1:** Demographic and clinical characteristics of the 286 participants.

Demographic and clinical characteristics	
Sex, n (%) women	255 (89.2)
Age, mean (SD) years	60.7 (6.2)
Body mass index, mean (SD) kg/m^2^	26.3 (4.6)
ACR criteria for hand OA, n (%)	267 (93.4)
ACR criteria for knee OA, n (%)^a^	176 (63.1)
WOMAC pain, median (IQR) (range: 0–20)	4.0 (0.3, 8.8)
Right knee pain last 6 weeks, n (%)	97 (33.9)
Left knee pain last 6 weeks, n (%)	87 (30.4)
Osteophyte sum score (both knees), median (IQR) (range: 0–24)	1.0 (0.0, 3.0)
Highest osteophyte score (both knees), n (%)	
• Grade 0	122 (42.7)
• Grade 1	77 (26.9)
• Grade 2	58 (20.3)
• Grade 3	29 (10.1)
GS synovitis sum score (both knees), median (IQR) (range: 0–6)	0.0 (0.0, 2.0)
Highest GS synovitis (both knees), n (%)	
• Grade 0	156 (54.5)
• Grade 1	75 (26.2)
• Grade 2	37 (12.9)
• Grade 3	18 (6.3)

a n ​= ​7 with missing information about ACR knee OA criteria. ACR=American College of Rheumatology; GS ​= ​Grey-scale; IQR ​= ​interquartile range; SD ​= ​standard deviation; WOMAC; Western Ontario McMaster Universities.

### 3.2 Comparison of ultrasound features between participants with and without clinical knee OA according to the ACR criteria

Data on clinical knee OA by the ACR criteria was available in 279 patients, of whom 176 (63.1 ​%) fulfilled the ACR criteria for clinical knee OA. Comparing participants with and without clinical knee OA according to the ACR criteria, we found minor between-group differences in sex (90.3 ​% vs. 87.4 ​% women) and age (mean 60.8 vs. 60.5 years), while higher BMI was found in those with clinical knee OA (mean 27.2 vs. 25.4 ​kg/m^2^). Participants who fulfilled the ACR criteria for clinical knee OA had more frequent and larger osteophytes than participants who did not fulfil the criteria (Table 2). Large (grade 3) osteophytes were mainly seen in patients who fulfilled the clinical knee OA criteria. Similar results were found when the right and left knee were analyzed separately with significantly more osteophytes in participants with vs. without clinical knee OA (p ​< ​0.001 and p ​= ​0.002 for right and left knee, respectively). No difference was found in the presence or severity of grey-scale synovitis in those with vs. without clinical knee OA (Table 2).

**Table 2:** Comparison of ultrasound-defined OA features among participants who did and did not fulfill the ACR classification criteria for clinical knee OA.

	Clinical knee OA (n = 176)	No clinical knee OA (n = 103)	P-value
Osteophyte sum score (both knees), median (IQR) (range 0–24)	2.0 (0.0, 5.0)	0.0 (0.0, 2.0)	<0.001
Highest osteophyte score (both knees), n (%)			
• Grade 0	60 (34.1)	61 (59.2)	<0.001
• Grade 1	51 (29.0)	25 (24.3)	
• Grade 2	39 (22.2)	16 (15.5)	
• Grade 3	26 (14.8)	1 (1.0)	
GS synovitis sum score (both knees), median (IQR) (range: 0–6)	0.0 (0.0, 2.0)	0.0 (0.0, 1.0)	0.61
Highest GS synovitis score (both knees), n (%)			0.67
• Grade 0	96 (54.5)	57 (55.3)	
• Grade 1	44 (25.0)	29 (28.2)	
• Grade 2	23 (13.1)	13 (12.6)	
• Grade 3	13 (7.4)	4 (3.9)	

ACR=American College of Rheumatology; GS = Grey-scale; IQR = interquartile range; OA = osteoarthritis.

### 3.2.1 Associations between ultrasound-detected OA features and knee pain

The sum score of osteophytes in both knees was statistically significantly associated with the severity of pain by the WOMAC questionnaire. Statistically significant associations with WOMAC pain were found for participants with at least one large osteophyte in their knee(s) (Table 3). On the other hand, we found no significant or clinically relevant associations between grey-scale synovitis and WOMAC pain (Table 3).

**Table 3:** Associations between ultrasound-defined OA features in the knees and WOMAC pain score (range: 0–20), adjusted for age, sex and BMI.

	Unstandardized beta (B) (95 ​% CI)
Osteophyte sum score (both knees, range: 0–24)	0.18 (0.03, 0.32)
Highest osteophyte score (both knees)	
• Grade 0	0.00 (ref)
• Grade 1	1.13 (−1.13, 2.40)
• Grade 2	0.67 (−0.74, 2.07)
• Grade 3	1.87 (0.04, 3.70)
GS synovitis sum score (both knees, range: 0–6)	0.03 (−0.33, 0.40)
Highest GS synovitis score (both knees)	
• Grade 0	0.00 (ref)
• Grade 1	0.24 (−0.99, 1.47)
• Grade 2	0.27 (−1.33, 1.87)
• Grade 3	0.46 (−1.73, 2.65)

BMI ​= ​body mass index; CI ​= ​confidence interval; GS ​= ​grey-scale; WOMAC=Western Ontario McMaster Universities Arthritis Index.

Increasing severity of ultrasound pathology was associated with higher odds of pain in the same knee both during the last 24 ​h and 6 weeks. For osteophytes, statistically significant associations with pain were found for small, moderate, and large osteophytes (Table 4). Only severe grey-scale synovitis was statistically significantly associated with pain in the same joint, while a borderline statistically significant association was found between moderate synovitis and knee pain during the last 24 ​h (Table 4).

**Table 4:** Associations between ultrasound pathology in the knees and pain in the same joint, adjusted for age, sex and BMI.

	OR (95 ​% CI) of pain previous 24 ​h	OR (95 ​% CI) of pain previous 6 weeks
Osteophyte sum score (range: 0–12)	1.33 (1.20, 1.48)	1.29 (1.15, 1.44)
Highest osteophyte score		
• Grade 0	1.00 (ref.)	1.00 (ref.)
• Grade 1	1.85 (1.20, 2.84)	1.79 (1.19, 2.68)
• Grade 2	2.77 (1.64, 4.70)	2.98 (1.76, 5.06)
• Grade 3	9.02 (4.04, 20.10)	6.50 (2.95, 14.30)
Highest GS synovitis score		
• Grade 0	1.00 (ref.)	1.00 (ref.)
• Grade 1	1.00 (0.66, 1.53)	0.95 (0.61, 1.47)
• Grade 2	1.67 (0.96, 2.91)	1.32 (0.78, 2.25)
• Grade 3	6.63 (2.26, 19.43)	4.32 (1.59, 11.71)

BMI ​= ​body mass index; CI ​= ​confidence interval; GS ​= ​grey-scale; OR ​= ​odds ratio.

## 4 Discussion

This study aimed to explore the frequency and severity of ultrasound-detected osteophytes and grey-scale synovitis in individuals meeting the ACR clinical criteria for clinical knee OA compared to those without clinical knee OA, as well as to examine the associations between ultrasound-detected osteophytes OA features and knee pain. In short, we showed that participants who fulfilled the ACR criteria for clinical knee OA had more frequent and larger osteophytes than those who did not fulfil the criteria, and osteophytes were significantly associated with WOMAC pain and pain in the same knee. Grey-scale synovitis was common in both groups, and only severe grey-scale synovitis was associated with pain in the same joint.

Ultrasound-detected knee osteophytes, but not grey-scale synovitis, were significantly more common in persons with vs. without clinical knee OA. First, it may reflect a dependency of bony enlargement in the ACR criteria. Second, synovitis is not necessarily specific to knee OA and may also occur in individuals without radiographic OA or symptomatic disease [29]. Grey-scale synovitis was almost equally distributed between those with and without clinical knee OA in our study, consistent with findings from prior studies with high prevalence of synovial abnormalities in elderly populations [29,30]. In a large general population sample, Jiang et al. demonstrated that nearly half of the participants (46.6 ​%) had knee effusion and 18.1 ​% had synovial hypertrophy, despite 61.2 ​% and 41.2 ​% of the joints with these positive ultrasound findings showed no radiographic OA [31]. Third, our control group consisted of individuals with hand OA, who were enrolled in a hospital-based setting. This is not a truly “healthy” population, as people with hand OA are at an increased risk for osteoarthritis in other joints, including the knees. This likely explains the relatively high prevalence of ultrasound-detected synovitis (44.7 ​%) and osteophytes (40.8 ​%) in participants without clinical knee OA.

Our results showed that ultrasound-detected osteophytes, but not grey-scale synovitis, were consistently associated with knee pain. These findings are consistent with previous studies demonstrating a link between osteophytes and knee pain using radiography, MRI, and ultrasound [3,32,33], while Oo et al. failed to demonstrate an association between ultrasound osteophytes and knee pain [17]. Knee joints with small to large osteophytes had two to nine times higher odds of having pain during the last 24 ​h in comparison with joints without osteophytes, highlighting the clinical relevance of ultrasound-detected osteophytes. A statistically significant association with pain severity by WOMAC was found for osteophyte sum score and large osteophytes. However, the strength of the association is of uncertain clinical value [34,35]. The differences in findings may be due to different outcome measures (pain presence versus severity), and we may speculate that pain severity may be more influenced by factors outside the joint than presence of pain. Furthermore, WOMAC does not differ between pain in the left or right knee, which may have affected the results.

In contrast, grey-scale synovitis showed no significant associations with WOMAC pain, while moderate to severe synovitis was associated with pain in the same joint, although statistically significant for severe synovitis only. Previous studies have shown conflicting results regarding the statistical significance of the association between synovitis and pain, which may be due to the relatively weak correlation as shown by Hall et al. [13]. Previous studies have also used different definitions of synovitis, focusing on effusion, synovial hypertrophy, or the combination of both. Oo et al. found statistically significant associations to pain severity for synovial hypertrophy, but not for effusion [17]. Since synovitis was assessed as a composite score of both hypertrophy and effusion in the Nor-Hand study, we were unable to assess these features separately in our analyses.

Importantly, the prevalence of synovial abnormalities, as also highlighted in recent reviews [5,36,37], remains high even in individuals without symptomatic OA. This aligns with OA now being considered as a whole-joint disease. Knee synovitis in individuals not fulfilling the criteria for clinical knee OA may represent early signs of OA. In line with these hypotheses, Felson et al. demonstrated that in knee joints free of radiographic knee OA at baseline, synovitis was a risk factor for developing OA within 84 months [38]. Whether treating synovitis may delay or prevent the onset of structural damage is currently not known. However, it should be noted that severe synovitis was more common in those with clinical knee OA and demonstrated strong and statistically significantly associations with pain in the same knee, suggesting that treating knees with severe synovitis may have a clinical benefit [39].

The strengths of our study include the definition of clinical knee OA using validated criteria in a large well-characterized study sample. Our cohort consisted of individuals with hand OA, a population known to be at increased risk of developing knee OA [28]. This provides a unique opportunity to examine ultrasound features of knee OA not only in those with established clinical disease but also in individuals without or at an early or subclinical stage of knee OA.

One notable limitation of our study is the moderate reliability observed for both osteophyte (weighted kappa ​= ​0.59) and synovitis (weighted kappa ​= ​0.48) scoring, which may have impacted the results. Other limitations include the cross-sectional design and the lack of other imaging data on the knee, such as MRI and radiographs, which limits our ability to study the concurrent validity of the ultrasound-detected features against other imaging outcomes. Additionally, the assessment of only suprapatellar synovitis may have led to underestimation of synovitis present elsewhere in the joint. However, synovitis at isolated parapatellar locations is likely mild and may not have clinical significance [40].

In conclusion, people fulfilling the ACR criteria for clinical knee OA had significantly more osteophytes by ultrasound than people without clinical knee OA, whereas no difference was found in the frequency and severity of synovitis. While knees with osteophytes of all sizes, including the small ones, were more likely painful than knees without osteophytes, a significant association between synovitis and pain was found in knees with severe synovitis only. These results may suggest that structural features are more important causes of pain than inflammation in knee OA.

## Author contributions

Conception and design (all authors); analysis and interpretation of the data (CHD, AM, IKH); drafting of the article (CHD, AM, IKH); critical revision of the article for important intellectual content (all authors); final approval of the article (all authors); provision of study materials or patients (IKH); statistical expertise (IKH); obtaining funding (IKH); administrative, technical, or logistical support (IKH); collection and assembly of data (all authors).

## Role of the funding source

The data collection of the Nor-Hand study is supported by grants from the South Eastern Norway Regional Health Authority, Simon Fougner Hartmanns Family foundation, Trygve Gythfeldt's research foundation, and Pahles foundation. The REMEDY center was funded by The Research Council of Norway (project number: 328,657). The funding sources had no role in the study design or in the collection, analysis, or interpretation of the data, the writing of the manuscript, or the decision to submit the manuscript for publication.

## Declaration of competing interest

HBH reports honorarium from Abbvie, Novartis, Lily and UCB, and advisory boards for Abbvie and Novartis, outside of the submitted work. IKH reports personal fees from Novartis, GSK and Grünenthal, and speaker honorarium from Abbvie, outside of the submitted work. CHD, AM, CMF and BSC report no competing interests.
